# AmberMDrun: A Scripting Tool for Running Amber MD in an Easy Way

**DOI:** 10.3390/biom13040635

**Published:** 2023-03-31

**Authors:** Zhi-Wei Zhang, Wen-Cai Lu

**Affiliations:** College of Physics, Qingdao University, Qingdao 266071, China

**Keywords:** scripting tool, automatically running Amber MD, Amber MD inputs, NVT and NPT balancing, ten step simulation preparation, MM-PB(GB)SA

## Abstract

MD simulations have been widely applied and become a powerful tool in the field of biomacromolecule simulations and computer-aided drug design, etc., which can estimate binding free energy between receptor and ligand. However, the inputs and force field preparation for performing Amber MD is somewhat complicated, and challenging for beginners. To address this issue, we have developed a script for automatically preparing Amber MD input files, balancing the system, performing Amber MD for production, and predicting receptor-ligand binding free energy. This script is open-source, extensible and can support customization. The core code is written in C++ and has a Python interface, providing both efficient performance and convenience.

## 1. Introduction

Molecular dynamics (MD) simulation has become a powerful tool in biomacromolecule simulation and computer-aided drug design, etc., to estimate receptor-ligand binding free energies and binding structures [1]. At present, there are several MD software packages, such as Amber, Gromacs and NAMD, etc [2,3,4,5]. Amber MD has achieved ultra-high performance due to rapid developments of GPU-based high-performance computing in recent years [6,7]. Generally, scientists prepare their own scripts for Amber MD simulations. The input files for the Amber MD mainly include (1) mdin, a control file for MD or energy minimization run, (2) prmtop, which contains molecular topology, force field and periodic box type, and (3) inpcrd (or a restart file, .rst7) which contains atomic coordinates, and (optionally) velocities and periodic box size [5]. The prmtop and inpcrd files can be generated by command-line utilities of AmberTools, including hydrogenation of protein, format conversion of small molecule coordinate file, and generation of force field parameter file. The Ambermdprep shell script is designed to balance most systems in MD simulations with simple and effective options [8], written in native Linux shell bash language. As a script in bash language, Ambermdprep is not easy for users to customize functions. Finally, users usually need to evaluate molecular mechanics Poisson–Boltzmann and generalized-Born surface area (MM-PB(GB)SA) binding free energies based on trajectory outputs. The commercial software Molecular Operating Environment (MOE) package implements a scripting method to call Amber, which can automatically run Amber MD by generating shell scripts, and setting up simulation procedures and parameters via a graphical interface. However, commercial software is expensive for most scientific researchers, and it is difficult for users to customize their own scripts. Therefore, a one-click scripting tool to run Amber and calculate MM-PB(GB)SA binding free energy is necessary. For some systems, Amber inputs are of high similarity. In this work, we developed a mixed script using C++ and Python with a one-click execution from processing protein and ligand structures, generation of small-molecule force field based on AmberTools, system balancing, MD simulation for production, and finally MM-PB(GB)SA calculation. The script is user-friendly to use due to software integration in Python, and an interface to the C++ is reserved to meet the user’s requirements for high performance. For users who are not very familiar with Amber MD, this scripting tool provides an automatic operation of the Amber MD. However, professional users can also set up parameters using detailed options or edit and modify the code.

## 2. Materials and Methods

Amber package has two major MD engines. Sander is a version of the original implementation, and Pmemd is a refactoring of sander. Pmemd introduced high-performance GPU computing to improve code performance.

### 2.1. Thermostat Methods

In MD simulations, it is essential to maintain system temperature through a thermostat method in which the temperature is directly controlled by adjusting the particle’s speeds. In the Berendsen thermostat [9], the temperature is controlled by an external thermal bath with a constant temperature, and the temperature of the system is maintained by absorbing or releasing energy from or to the thermal bath. When the system is far from equilibrium, the temperature regulation is very efficient, but the kinetic energies of particles do not strictly follow a Boltzmann distribution. Therefore, the Berendsen thermostat can be applied in the heating phase, but not in the pre-equilibration phase. When the system has been fully balanced, a weak coupling constant (such as 10 ps) will be used, so that the Berendsen thermostat can be used in the production (sampling) stage. Note that the Berendsen thermostat is not suitable for implicit solvent simulation, because it cannot help to maintain a constant temperature by collisions with solvent molecules. In the Langevin thermostat [10,11], the speed and accuracy of heat bath to control temperature are at an intermediate level, it adjusts the speeds of particles by virtual random collisions, disturbing normal evolution of the system and weakening velocity correlations among particles. The Langevin thermostat strictly follows canonical ensemble attributes, which does not affect the ergodicity of each state. The Langevin dynamics is suitable for simulating thermodynamic properties, such as binding free energy, but not kinetic properties. It can be especially useful in implicit-solvent calculations where it can mimic the forces due to solvent. However, when running all-atom MD with explicit solvent, introducing friction and random forces can have an undesirable impact on the dynamics. On the other hand, the only absolutely correct way to get the ”pure“ dynamics due to acting potential forces would be running it in the NVE mode using a very large periodic water box or, better, a very large water droplet (to be free from the artifacts due to periodicity). While introducing a thermostat, such as the Langevin thermostat, can perturb the motion due to potential forces, it is often necessary to do so to accurately simulate a system at a desired temperature. It’s essential to choose the appropriate level of friction and random forces based on the desired temperature and simulation conditions, which can vary depending on the research question and system being studied. Notably, the Langevin thermostat can still produce accurate results even when water friction is scaled down by a factor of 100, as demonstrated in Kleinerman et al. [12]. Extended Lagrangian methods can overcome the shortcomings of the above methods using time inversion. A representative method is the Nosé–Hoover thermostat [13], which is the simplest method with time reversibility. However, when the system is far from equilibrium, temperature oscillation is large and slow to converge. Since Nosé–Hoover implements the thermodynamics of the canonical ensemble and can approximate the true kinetic behavior, the Nosé–Hoover thermostat is applicable in the equilibrium sampling phase, rather than in the heating phase. It is worth mentioning that the Nosé–Hoover thermostat may have morbid behavior in specific systems [14,15]. In recent years, some improved methods have been developed, such as optimized isokinetic Nose-Hoover chain (OIN) and stochastic isokinetic Nosé–Hoover RESPA integrator schemes [16,17].

### 2.2. Barostat Methods

In NPT systems, it is necessary to use a barostat to control the system pressure. Mostly used methods include the Berendsen, Nosé–Hoover and Parrinello–Rahman barostats [18]. When the system is far from equilibrium, the Berendsen barostat is efficient in controlling the pressure and suitable for the initial pressure relaxation of the system, but not suitable for balanced sampling since it does not achieve sampling with a canonical distribution. The Nosé–Hoover and Parrinello–Rahman methods are more applicable to pressure control of balanced sampling than the Berendsen method [13,18]. Monte Carlo (MC) method is also useful, but its efficiency is relatively low [19]. Thus, MC is more suitable for the sampling stage.

### 2.3. Ten Step Simulation Preparation Protocol

System balancing consists of ten steps in simulation preparation [8]. MIN1 is a 1000-step steepest descent (SD) minimization where heavy atoms of the acceptor are constrained. MIN2 is an MD simulation at 15 ps with a time step of 1 fs, with confinement energy of 5.0 kcal/mol for heavy atoms of macromolecules and involving H atoms. MIN3 is similar to MIN1 except that the heavy atom binding force of the macromolecule is changed to 2.0 kcal/mol. MIN4 is a 1000-step gradient descent energy optimization with confinement energy of 0.1 kcal/mol for large heavy atoms. MIN5 is the SD minimization of 1000 steps without any constraints. MIN6 is a 5 ps MD with a time step of 1 fs for NPT systems with energies of 1.0 kcal/mol on heavy atoms and bonds involving H atoms in macromolecules. MIN7 is similar to MIN6 except that the energy on the heavy atoms of the macromolecule is 0.5 kcal/mol. MIN8 is an MD simulation with a time step of 1 fs in the NPT ensemble and a time of 10 ps. In MIN8, non-hydrogen backbone atoms of proteins and nucleic acid residues and heavy atoms of macromolecules are constrained. MIN9 is an NPT simulation at 10 ps with a time step of 2 fs without any constraints. MIN10 is a final density stabilized MD.

### 2.4. AM1-BCC Method

AM1-BCC is an accurate and efficient method for computing atomic charges that are used to represent the electron distribution of molecules. The AM1-BCC method combines the Austin Model 1(AM1) semi-empirical method to generate the initial guess charges and the bond-charge-increment (BCC) scheme to correct the charges [20,21].

In the AM1-BCC methodology, the atomic charge($q_{j}$) of a specific atom(j) is determined through the combination of two separate terms Equation (1). The initial value of $q_{j}^{pre}$ is determined through a rapid precharge process that takes into account the majority of the chemical aspects, but this value alone is insufficient to be utilized in Equation (1) for simulations of materials in their condensed phase. The AM1 method utilizes atomic charges that are averaged based on bond symmetry, and these charges are indeed used for the calculations. However, the electrostatic potential (ESP) obtained from the AM1 method is not accurate enough to match the ESP obtained from more advanced methods such as HF/6-31G∗. Therefore, a correction term, $q_{j}^{corr}$, is added in Equation (1) to adjust the AM1 atomic charges and closely reproduce the HF/6-31G∗ ESP. $q_{j}^{corr}$ is defined in Equation (2). Where pα is the bond charge correction (BCC) for bond type α. The bond connectivity template matrix T is a mathematical tool used in the calculation of atom-centered charges from bond charges. It maps the BCCs for each bond type in a molecule onto the corresponding atom types.

Overall, AM1-BCC is a robust method that can provide reliable partial atomic charges for a wide range of molecular systems. It is widely used in drug discovery and molecular simulations to examine the interaction of molecules with proteins and other biological targets [22].

(1) $$q_{j}=q_{j}^{pre}+q_{j}^{corr}$$

(2) $$q_{j}^{corr}=\sum_{\alpha =1}^{\gamma }T_{j\alpha p\alpha }$$

### 2.5. MM-PB(GB)SA Calculations

The Molecular Mechanics/Poisson Boltzmann (Generalized Born) Surface Area (MM/PB (GB) SA) method is often used to calculate the binding free energy of non-covalently bound complexes. The binding free energy between ligand and receptor is the relative free energy of complex concerning the sum of free energies between receptor and ligand, which is calculated by Equation (3). The molecular mechanics PoissonBoltzmann surface area (MM-PBSA) and molecular mechanics generalized Born surface area (MM-GBSA) methods for binding free energies were developed by Kollman et al. [23,24,25]. MM-PBSA is an efficient method for the calculation of free energies of diverse molecular systems [26]. The PBSA method is more time-consuming, and the GBSA is an analytical approximation to the PBSA method. The MM-PBSA and MM-GBSA free energies of solvation terms, $\Delta G_{sol}^{complex}$, $\Delta G_{sol}^{receptor}$, $\Delta_{sol}^{ligand}$, include electronic interaction (polar contribution) calculated by solving the Poisson–Boltzmann equation or the generalized Born equation and the hydrophobic interaction (non-polar contribution) Equations (3)–(6). The solvation interaction includes the electronic (polar) interaction $\Delta G_{GB/PB}$ with electrostatic potentials calculated by solving the Poisson–Boltzmann or generalized Born equation, and hydrophobic (non-polar) interaction $\Delta G_{non-polar}$ calculated empirically by solvent-accessible surface area (SASA) [27]. where γ and b are constants that can be determined by fitting to the experimental alkane transfer free energies versus SASA [27,28,29]. In order to obtain the MM-PB(GB)SA binding free energy, two methods, the single-trajectory protocol (STP) and the multi-trajectory protocol (MTP), can be used. The STP method extracts receptor and ligand trajectories from the complex, while the MTP method uses multiple trajectories for the complex, receptor and ligand [30,31].

(3) $$\Delta G_{bind}=\Delta G_{bind,gas}+\Delta G_{sol}^{complex}-\left(\Delta G_{sol}^{receptor}+\Delta G_{sol}^{ligand}\right)$$

(4) $$\Delta G_{sol}=\Delta G_{polar}+\Delta G_{non-popar}=\Delta G_{PB/GB}+\Delta G_{non-popar}$$

(5) $$\Delta G_{non-popar}=\gamma \times \Delta SASA+b$$

(6) $$\Delta G_{bind,gas}=\Delta E_{MM}-T\Delta S=\Delta E_{bonded}+\Delta E_{ele}+\Delta E_{vdW}-T\Delta S$$

## 3. Results

The script, AmberMDrun, is mainly written in C++, with Python binding provided by Pybind11. The C++ code ensures high performance, while the Python binding allows for ease of use for users. The script’s default parameter values have been carefully set, but users have the flexibility to customize their own MD simulations by making adjustments to only a few parameters. Additionally, the script’s good object-oriented design makes it possible for users who want to extend the package to do so easily.

In the following sections, we will provide an overview of the script’s main flows and demonstrate its effectiveness in MD simulations.

### 3.1. Install

The package requires GCC version 9.3 or higher and Python version 3.6 or higher to run. In order to install AmberMDrun and use the scripts, one can use either of the following commands: conda install ambermdrun -c zjack or pip3 install AmberMDrun.

### 3.2. Core Classes in the AmberMDrun

Systeminfo class Check whether pmemd.cuda is available, and then generate commands to run the amber MD job with pmemd.cuda or sander. Furthermore, the number of receptor residues will be obtained for positional restraints of the receptor in the later system balancing.

MIN class Perform energy minimization. Maxcyc (the maximum number of cycles of minimization) and ntmin (flag for the method of minimization), etc., are set up (Table 1).

NVT class Perform an NVT simulation. The thermostat method and nstlim, etc., are set up (Table 1).

NPT class Perform an NPT simulation. Barostat and thermostat methods and nstlim, etc., are set up (Table 1).

When MD completes, the track file in NetCDF format is obtained for subsequent analyses, implemented by the class MM-PBSA or MM-GBSA. A simple example can show how simple it is to run the script, with the steps of Inputs ⇒Systeminfo ⇒ Min ⇒ NVT ⇒ NPT ⇒ MD ⇒ MM-PBSA or MM-GBSA.

### 3.3. Example

**Figure 1:**
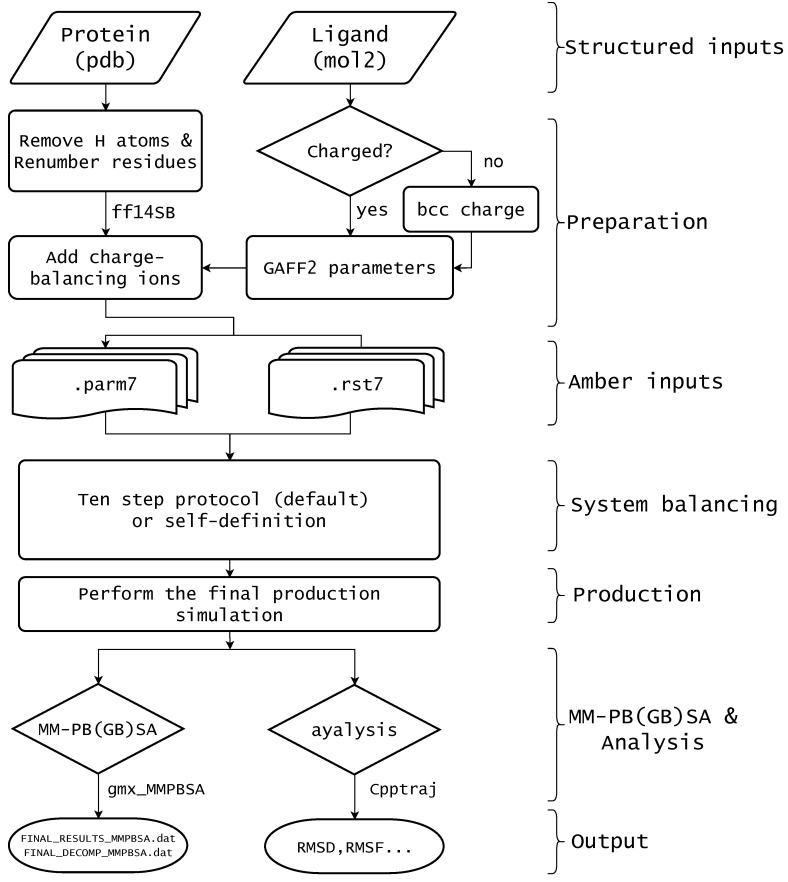
Workflow of the script AmberMDrun, which depends on AmberTools, Amber (optional) [5], and gmx_MMPBSA [36]. The ten-step protocol [8] is set as the default system balancing method, and users can also define their own balancing method. Using gmx_MMPBSA, MM-PBSA or MM-GBSA binding free energies are estimated.

In biomacromolecule studies and common drug-aided design, predicting MM-PB(GB)SA is often called for. In this study, we provide a programming tool named AmberMDrun, which offers a simple and user-friendly script implementation, including automatic generation of Amber inputs, system balancing, MD simulations for production, and binding-free energy calculations. The process is shown in Figure 1. As an example, we tested the system of raloxifene binding to the estrogen receptor in Amber’s tutorial. In the AmberMDrun script, only the protein and ligand coordinate files are necessary. Typically, when conducting MMPBSA calculations for most protein-ligand complexes, it is best to use the complex structure after docking. So, after docking, it is recommended to save the protein’s PDB file and the ligand’s mol2 file separately. However, if only the PDB files of the protein and ligand are provided, the ambermdrun script offers a straightforward splitting program. It’s important to note that the user must always verify the accuracy of the ligand structure, even after docking. The Amber input files are generated using tleap and Acpype. The force field parameterization of the ligand was carried out using Acpype and the general force field of gaff2 is used for the ligand, the small molecule charges were calculated by AM1-BCC method when they lacked [20,21,22,32,33,34]. The force field for the protein is amber14SB [35]. System balancing was automatically executed by (default) the ten-step protocol [8]. Then, a 50 ns MD simulation for production was carried out, and finally, the binding free energy between receptor and ligand was obtained by calling gmx_MMPBSA [36]. The gmx_MMPBSA was originally intended to provide convenience for Gromacs users to call the MMPBSA.py script [37], In our AmberMDrun script, it is applied to calculate the binding free energies of receptor-ligand complexes.

**Figure 2:**
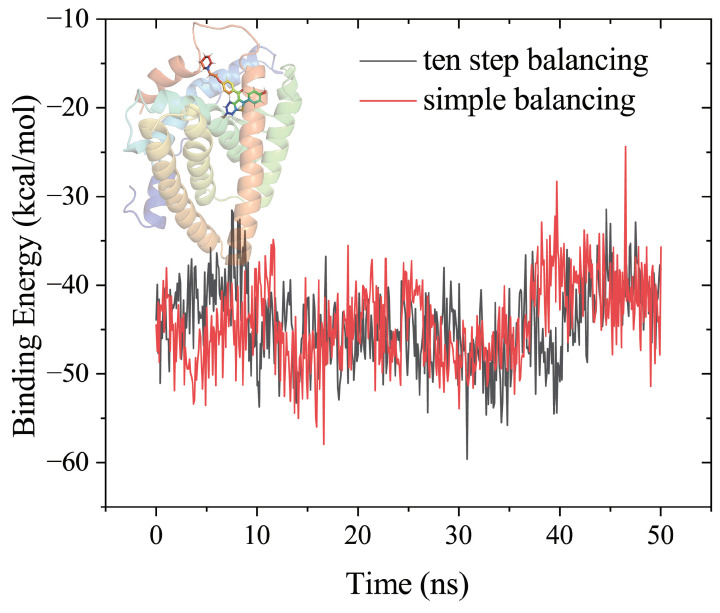
Comparison of the MM-PBSA results from 50 ns MD simulations of the raloxifen-estrogen receptor, using the ten step balancing or simple balancing in the Amber tutorial, using the AmberMDrun script.

Figure 2 showed the MM-PBSA binding free energies estimated by 50 ns MD simulations for production after ten-step balancing or simple balancing in the Amber tutorial. The results obtained by the two balancing procedures are almost the same for this system.

**Figure 3:**
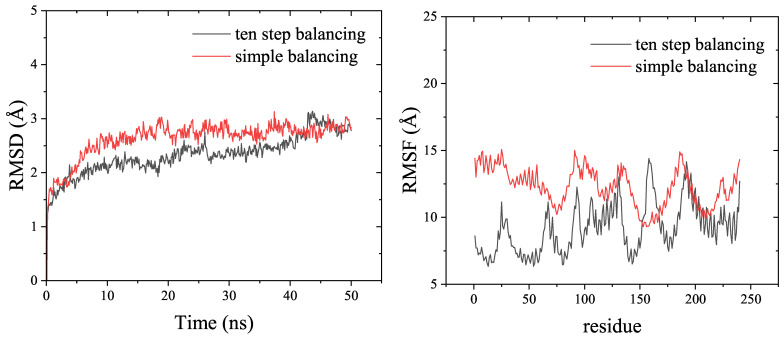
Comparison of the RMSD and RMSF results from 50 ns MD simulations of the raloxifen-estrogen receptor, by the ten-step balancing or simple balancing in the Amber tutorial, using the AmberMDrun script.

RMSD (Root Mean Square Deviation) and RMSF (Root Mean Square Fluctuation) are usually used to determine the stability of the recaptor-ligand complex in MD simulations. According to Figure 3, both the ten-step balancing and simple balancing methods produced stable raloxifene-estrogen receptor complexes during the entire simulation time. Overall, the RMSD and RMSF values obtained with the ten-step balancing method were lower than those obtained with the simple balancing method for the raloxifene-estrogen receptor example presented in the Amber tutorial [38].

We also tested the example of the raloxifene-estrogen receptor complex in the Amber tutorial using the AmberMDrun script. We compared three results in Table 2: (1) from the tutorial, (2) repeating with the simulation balancing preparation in the Amber tutorial, and (3) ten-step balancing [8]. It can be seen that results (2) and (3) are almost the same and the binding free energy of result (1) is slightly higher than those of (2) and (3). Using the scripting tool AmberMDrun, only a one-click operation can complete the complicated job “mmpbsa -p protein.pdb -m mol.mol2 –ns 50”, which is automatically executed, realizing our goal to run Amber easily, like openmm that can realize its functions through Python [39].

## 4. Discussion

The AmberMDrun is an integrated script written by Python and C++, which is designed for automatically generating input files by processing structures of proteins and small molecules, producing small-molecule force fields, balancing system, running MD simulations, and achieving MM-PB(GB)SA binding free energies. This script is a lightweight and convenient package that can be easily incorporated into molecular simulation studies to expedite progress.

For enhanced sampling, such as GaMD, users can inherit and implement their own GaMD classes from the existing NPT class. However, many users are not familiar with Python or C++, which can be a barrier to usage. In the future, one direction of study could be to develop unique classes for various enhanced sampling dynamics. Another option could be to simplify the parameters or introduce a graphical interface to make it more accessible to users. Additionally, re-writing the main components of tleap in C++ could result in a more convenient tool for generating topological coordinate files, utilizing the user-friendly syntax and graphical interface offered by Python. In addition, using a web server instead of a graphical interface is also a good option, as described in the article [40].

## Figures and Tables

**Table 1:** Default values of some parameters in the AmberMDrun.

	MIN	NVT	NPT
Params	imin = 1	imin = 0	imin = 0
		ntb = 1	ntb = 2
temp = 298.15 ^1^	✘	✔	✔
cut = 8.0 ^2^	✔	✔	✔
ntpr = 50 ^3^	✔	✔	✔
ntwr = 500 ^4^	✔	✔	✔
ntwx = 500 ^5^	✔	✔	✔
maxcyc = 1000 ^6^	✔	✘	✘
ncyc = 10 ^7^	✔	✘	✘
ntmin = 10 ^8^	✔	✘	✘
nstlim = 5000 ^9^	✘	✔	✔
dt = 0.002 ^10^	✘	✔	✔
irest = False ^11^	✘	✔	✔
tautp = 1.0 ^12^	✘	✔	✔
taup = 1.0 ^13^	✘	✘	✔
gamma_ln = 5.0 ^14^	✘	✔	✔
nscm = 0 ^15^	✘	✔	✔
ntc = 2 ^16^	✘	✔	✔
ntf = 2 ^17^	✘	✔	✔
thermostat ^18^	✘	✔	✔
barostat ^19^	✘	✘	✔
igamd = false ^20^	✘	✘	✘

^1^ simulated temperature. ^2^ specify the nonbonded cutoff, in Angstroms. ^3^ print in human-readable form to files. ^4^ restart file input frequency. ^5^ coordinate file input frequency. ^6^ The maximum number of cycles of minimization. ^7^ the method of minimization will be switched from steepest descent to conjugate gradient after ncyc cycles. ^8^ flag for the method of minimization ^9^ Number of MD-steps to be performed. ^10^ The time step. ^11^ Flag to restart a simulation. ^12^ Time constant. ^13^ Pressure relaxation time. ^14^ The collision frequency. ^15^ Flag for the removal of translational and rotational center-of-mass (COM) motion at regular intervals (default is 1000) ^16^ Flag for SHAKE to perform bond length constraints. ^17^ Force evaluation. ^18^ Thermostat selection. ^19^ Barostat selection. ^20^ Whether to execute gamd.

**Table 2:** Comparison of MM-PBSA and component results from 2 ns MD simulations of the raloxifen-estrogen receptor complex.

Result	Δ *vdW*	$\Delta E_{\mathrm{EL}}$	$\Delta E_{\mathrm{PB}}$	$\Delta E_{\mathrm{NPOLAR}}$	$\Delta E_{\mathrm{GAS}}$	$\Delta E_{\mathrm{SOL}}$	Δ *TOTAL*
Tutorial ^1^	−56.29	−38.03	57.64	−5.04	−94.32	52.60	−41.73
Simple balancing ^2^	−55.48	−41.00	53.79	−5.58	−96.48	48.21	−48.27
Ten step balancing ^3^	−58.62	−36.00	54.00	−5.70	−94.63	48.30	−46.32

^1^ results in the Amber tutorial. ^2^ results with simple balancing in the Amber tutorial. ^3^ results with ten step balancing.

## Data Availability

The source codes and data are available at https://github.com/9527567/AmberMDrun accessed on 27 March 2023.

## Abbreviations

The following abbreviations are used in this manuscript:

ESP	electrostatic potential
AM1	Austin Model 1
BCC	Bond charge correction
ΔvdW	van der Waals energy
$\Delta E_{EL}$	Electrostatic energy
$\Delta E_{PB}$	Polar solvation energy
$\Delta E_{NPOLAR}$	Non-polar solvation energy
$\Delta E_{GAS}$	Total gas phase free energy
$\Delta E_{SOL}$	Total solvation free energy
$\Delta E_{TOTAL}$	Total energy

## References

[1] Maginn E.J. (2009). From discovery to data: What must happen for molecular simulation to become a mainstream chemical engineering tool. AIChE J..

[2] Pronk S., Páll S., Schulz R., Larsson P., Bjelkmar P., Apostolov R., Shirts M.R., Smith J.C., Kasson P.M., van der Spoel D. (2013). GROMACS 4.5: A high-throughput and highly parallel open source molecular simulation toolkit. Bioinformatics.

[3] Phillips J.C., Braun R., Wang W., Gumbart J., Tajkhorshid E., Villa E., Chipot C., Skeel R.D., Kalé L., Schulten K. (2005). Scalable molecular dynamics with NAMD. J. Comput. Chem..

[4] Phillips J.C., Hardy D.J., Maia J.D.C., Stone J.E., Ribeiro J.a.V., Bernardi R.C., Buch R., Fiorin G., Hénin J., Jiang W. (2020). Scalable molecular dynamics on CPU and GPU architectures with NAMD. J. Chem. Phys..

[5] Case D., Aktulga H., Belfon K., Ben-Shalom I., Berryman J., Brozell S., Cerutti D., Cheatham T., Cisneros G., Cruzeiro V.W.D. (2022). Amber 2022.

[6] Götz A.W., Williamson M.J., Xu D., Poole D., Le Grand S., Walker R.C. (2012). Routine Microsecond Molecular Dynamics Simulations with AMBER on GPUs. 1. Generalized Born. J. Chem. Theory Comput..

[7] Salomon-Ferrer R., Götz A.W., Poole D., Le Grand S., Walker R.C. (2013). Routine Microsecond Molecular Dynamics Simulations with AMBER on GPUs. 2. Explicit Solvent Particle Mesh Ewald. J. Chem. Theory Comput..

[8] Roe D.R., Brooks B.R. (2020). A protocol for preparing explicitly solvated systems for stable molecular dynamics simulations. J. Chem. Phys..

[9] Berendsen H.J.C., Postma J.P.M., van Gunsteren W.F., DiNola A., Haak J.R. (1984). Molecular dynamics with coupling to an external bath. J. Chem. Phys..

[10] Uberuaga B.P., Anghel M., Voter A.F. (2004). Synchronization of trajectories in canonical molecular-dynamics simulations: Observation, explanation, and exploitation. J. Chem. Phys..

[11] Sindhikara D.J., Kim S., Voter A.F., Roitberg A.E. (2009). Bad Seeds Sprout Perilous Dynamics: Stochastic Thermostat Induced Trajectory Synchronization in Biomolecules. J. Chem. Theory Comput..

[12] Kleinerman D.S., Czaplewski C., Liwo A., Scheraga H.A. (2008). Implementations of Nosé–Hoover and Nosé–Poincaré thermostats in mesoscopic dynamic simulations with the united-residue model of a polypeptide chain. J. Chem. Phys..

[13] Posch H.A., Hoover W.G., Vesely F.J. (1986). Canonical dynamics of the Nosé oscillator: Stability, order, and chaos. Phys. Rev. A.

[14] Lingenheil M., Denschlag R., Reichold R., Tavan P. (2008). The “Hot-Solvent/Cold-Solute” Problem Revisited. J. Chem. Theory Comput..

[15] Basconi J.E., Shirts M.R. (2013). Effects of Temperature Control Algorithms on Transport Properties and Kinetics in Molecular Dynamics Simulations. J. Chem. Theory Comput..

[16] Omelyan I., Kovalenko A. (2013). Multiple time step molecular dynamics in the optimized isokinetic ensemble steered with the molecular theory of solvation: Accelerating with advanced extrapolation of effective solvation forces. J. Chem. Phys..

[17] Chen P.Y., Tuckerman M.E. (2018). Molecular dynamics based enhanced sampling of collective variables with very large time steps. J. Chem. Phys..

[18] Bussi G., Zykova-Timan T., Parrinello M. (2009). Isothermal-isobaric molecular dynamics using stochastic velocity rescaling. J. Chem. Phys..

[19] Vitalis A., Pappu R.V., Wheeler R.A. (2009). Chapter 3 Methods for Monte Carlo Simulations of Biomacromolecules. Annual Reports in Computational Chemistry, Chapter 3 Methods for Monte Carlo Simulations of Biomacromolecules.

[20] Jakalian A., Bush B.L., Jack D.B., Bayly C.I. (2000). Fast, efficient generation of high-quality atomic charges. AM1-BCC model: I. Method. J. Comput. Chem..

[21] Jakalian A., Jack D.B., Bayly C.I. (2002). Fast, efficient generation of high-quality atomic charges. AM1-BCC model: II. Parameterization and validation. J. Comput. Chem..

[22] He X., Man V.H., Yang W., Lee T.S., Wang J. (2020). A fast and high-quality charge model for the next generation general AMBER force field. J. Chem. Phys..

[23] Srinivasan J., Cheatham T.E., Cieplak P., Kollman P.A., Case D.A. (1998). Continuum Solvent Studies of the Stability of DNA, RNA, and Phosphoramidate-DNA Helices. J. Am. Chem. Soc..

[24] Kollman P.A., Massova I., Reyes C., Kuhn B., Huo S., Chong L., Lee M., Lee T., Duan Y., Wang W. (2000). Calculating Structures and Free Energies of Complex Molecules: Combining Molecular Mechanics and Continuum Models. Account. Chem. Res..

[25] Srinivasan J., Miller J., Kollman P.A., Case D.A. (1998). Continuum Solvent Studies of the Stability of RNA Hairpin Loops and Helices. J. Biomol. Struct. Dyn..

[26] Homeyer N., Gohlke H. (2012). Free Energy Calculations by the Molecular Mechanics Poisson–Boltzmann Surface Area Method. Mol. Inform..

[27] Sitkoff D., Sharp K.A., Honig B. (1994). Accurate Calculation of Hydration Free Energies Using Macroscopic Solvent Models. J. Phys. Chem..

[28] Connolly M.L. (1983). Analytical molecular surface calculation. J. Appl. Crystallogr..

[29] Rastelli G., Rio A.D., Degliesposti G., Sgobba M. (2010). Fast and accurate predictions of binding free energies using MM-PBSA and MM-GBSA. J. Comput. Chem..

[30] Wang E., Sun H., Wang J., Wang Z., Liu H., Zhang J.Z.H., Hou T. (2019). End-Point Binding Free Energy Calculation with MM/PBSA and MM/GBSA: Strategies and Applications in Drug Design. Chem. Rev..

[31] Lee M.S., Olson M.A. (2006). Calculation of Absolute Protein-Ligand Binding Affinity Using Path and Endpoint Approaches. Biophys. J..

[32] Sousa da Silva A.W., Vranken W.F. (2012). ACPYPE—AnteChamber PYthon Parser interfacE. BMC Res. Notes.

[33] Wang J., Wolf R.M., Caldwell J.W., Kollman P.A., Case D.A. (2004). Development and testing of a general amber force field. J. Comput. Chem..

[34] Wang J., Wang W., Kollman P.A., Case D.A. (2006). Automatic atom type and bond type perception in molecular mechanical calculations. J. Mol. Graph. Model..

[35] Maier J.A., Martinez C., Kasavajhala K., Wickstrom L., Hauser K.E., Simmerling C. (2015). ff14SB: Improving the Accuracy of Protein Side Chain and Backbone Parameters from ff99SB. J. Chem. Theory Comput..

[36] Valdés-Tresanco M.S., Valdés-Tresanco M.E., Valiente P.A., Moreno E. (2021). gmx_MMPBSA: A New Tool to Perform End-State Free Energy Calculations with GROMACS. J. Chem. Theory Comput..

[37] Miller B.R.I., McGee T.D.J., Swails J.M., Homeyer N., Gohlke H., Roitberg A.E. (2012). MMPBSA.py: An Efficient Program for End-State Free Energy Calculations. J. Chem. Theory Comput..

[38] McGee D., Miller B.J.S. (2009). Python Script MMPBSA.py. https://ambermd.org/tutorials/advanced/tutorial3/py_script/index.php/.

[39] Eastman P., Friedrichs M.S., Chodera J.D., Radmer R.J., Bruns C.M., Ku J.P., Beauchamp K.A., Lane T.J., Wang L.P., Shukla D. (2013). OpenMM 4: A Reusable, Extensible, Hardware Independent Library for High Performance Molecular Simulation. J. Chem. Theory Comput..

[40] Zhiyong C., Zhang Z., Zhou T., Zhou X., Zhang Y., Meng H., Wang W., Liu Y. (2022). A TastePeptides-Meta system including an umami/bitter classification model Umami_YYDS, a TastePeptidesDB database and an open-source package Auto_Taste_ML. Food Chem..

